# Evidence-Informed Deliberative Processes for UHC: Progress, Potential and Prudence: Comment on "Evidence-Informed Deliberative Processes for Health Benefit Package Design – Part II: A Practical Guide"

**DOI:** 10.34172/ijhpm.2022.7541

**Published:** 2023-02-13

**Authors:** Unni Gopinathan

**Affiliations:** Global Health Cluster, Division for Health Services, Norwegian Institute of Public Health, Oslo, Norway.

**Keywords:** Inclusiveness, Fairness, Legitimacy, Deliberation, Health Technology Assessment, Priority-Setting

## Abstract

In their recent article on evidence-informed deliberative processes (EDPs) for health benefit package decisions, Oortwijn et al examine how the different steps of EDP play out in eight countries with relatively mature institutions for using health technology assessment (HTA). This commentary examines how EDP addresses stakeholder involvement in decision-making for equitable progress towards universal health coverage (UHC). It focuses on the value of inclusiveness, the need to pay attention to trade-offs between desirable features of EDP and the need to broaden the scope of processes examined beyond those specifically tied to producing and using HTAs. It concludes that EDPs have contributed to significant progress for health benefit design decisions worldwide and holds much potential in further application. At the same time, this commentary calls for prudence: investments in EDPs should be efficiently deployed to enhance the pre-existing legislative, institutional and political framework that exist to promote fair and legitimate healthcare decisions.

## Introduction

 The goal of fair and legitimate processes when determining health benefit packages for universal health coverage (UHC) has gained significant research and policy attention over the past two decades. The seminal work of Daniels and Sabin on the accountability for reasonableness (A4R) Framework, initially developed in the context of health insurance decisions in the United States,^1^ paved the way for thinking systematically about the features of decision-making that promote fairness and legitimacy. The conditions for a fair and legitimate process proposed by A4R have since been applied in settings ranging from Mexico,^2^ United Kingdom,^3^ and Tanzania.^4^ Motivated by similar questions about how features of the decision-making process can promote procedural values like legitimacy and trust, other frameworks like the Kapiriri and Martin framework have also been produced.^5^

 Informed by these contributions, “evidence-informed deliberative process” (EDP) has emerged as a framework to guide fair and legitimate priority-setting for health benefit package design and the use of health technology assessments (HTAs). Implementation of EDP is composed of six steps: (1) installing an advisory committee; (2) defining decision criteria; (3) selecting health technologies for HTA; (4) Scoping, assessing and appraising; (5) Communication and appeal and; (6) Monitoring and evaluation. In their recent article, Oortwijn et al examine, informed by a review of publicly available documentation, how these different steps of EDP play out in eight countries with relatively mature institutions for HTA-informed processes.^6^

 Chiefly, this commentary will examine how EDP addresses stakeholder involvement in decision-making for equitable progress towards UHC, focusing on the value of inclusiveness. Inclusiveness is a fundamental value in democratic theory for securing sound deliberative processes.^7,8^ In the context of HTA-informed processes, inclusiveness is key to securing the robust inclusion of social values in the decision-making process. Yet if EDP is meant to represent a legitimate process for determining health benefits, the main approaches envisaged for stakeholder involvement may need critical re-examination. Tied to the focus on inclusiveness, the commentary further examines two aspects. First, the need to pay attention to trade-offs between desirable features of EDP. For example, strict management of conflict of interest may prevent inclusive deliberation. Moreover, full transparency, such as using open meetings, does not necessarily align well with a sound deliberative process. Second, public and political arenas where the redistributive nature of UHC policies are debated goes beyond structured and systematic HTA-driven processes.^9^ Evaluations of fair and legitimate decision-making must therefore take a broad approach to the scope of processes studied.

## Inclusiveness

 Inclusiveness is a fundamental value for democratic processes and a central concept for guiding fair and legitimate decision-making. It represents a standard with distinct features that have implications for how EDPs are designed and implemented. First and foremost, inclusiveness is about securing *representation* in the decision-making process by all relevant voices and interests who are affected by the decision.^7,8^ Accordingly, inclusiveness implies *diversity*, in that a wide range of views that are expressed by members of the public are reflected in the decision-making process. This is especially important for priority-setting processes in healthcare, which tend to affect interests across the population — patients with the stake in the treatment option, their families as well as populations who will bear the opportunity cost. Second, achieving inclusiveness is deeply tied to political *equality*: that anyone affected by the decision, regardless of social, economic or political status, should have a say in the decision-making process and that their arguments are given equal consideration.^7^ Tied to this point, securing representation and diversity requires attention to removing barriers to participation.^10^

 In the EDP as described by Oortwijn et al,^6^ “inclusiveness” is primarily achieved by ‘stakeholder involvement’—which is presented as one of four key ways of achieving a legitimate process. In the context of EDPs, ‘stakeholder participation’ can be achieved by direct membership and voting power in the advisory committees of HTA bodies. Oortwijn et al distinguish between two types of members: ‘experts’ and ‘non-experts,’ where the latter are members representing general interests of patients or industry. Beyond the deliberative process that occur in the advisory committee, Oortwijn et al describe three approaches for involvement. First, stakeholder participation by actors representing interests or expertise pertaining to the specific health technology in question. Second, consultations where stakeholders can express opinions through verbal comments during meetings or in written formats. Third, communication by which stakeholders are informed about the processes or decisions but not otherwise engaged in a mutual exchange. Cutting across these approaches is the need to pay attention to financial, social, cultural and administrative barriers that typically prevent participation of under-represented groups.^10^ A key question is whether the conventional focus on including patients when facilitating stakeholder involvement fall short of the deliberative ideals for fairness and legitimacy.^11^ Moreover, is there scope for more ambitious approaches for securing inclusiveness in the context of implementing EDP? A strategy promoted by deliberative democratic theorists and practitioners is the use of mini-publics, citizen juries and other formats for securing inclusive deliberation.^12^ Such approaches require considerable time and investment; it might not be reasonable to expect such ambitious level of public involvement for every health technology that is being considered, especially in low-resource settings where public administrative capacity is limited. However, such approaches can be valuable for eliciting public views and preferences about fundamental questions and support the foundations of EDP and similar processes, like substantive criteria that ought to be considered when determining health benefits.^13^

## Trade-offs

 There can be conflicts between the procedural features deemed important for fair and legitimate decision-making processes. Transparency is generally seen as desirable to HTA processes and is one of the key conditions of A4R (in this framework described as “publicity”). Yet implementing transparency involves benefits and burdens to the HTA process. For example, while open meetings can enable members of the public and other stakeholders to listen in and critically evaluate the arguments for different positions, such level of transparency can also constrain participants from freely expressing their views and thereby prevent a sound deliberative process. In their empirical assessment, Oortwijn et al report that the advisory committee of one country (Brazil) held open meetings, three countries used closed door discussion when required (Germany, UK, Scotland) and four committees held closed meetings (France, Thailand, Canada, and Australia).^12^ The EDP guide stipulate that transparency achieved through open meetings may improve the recommendations, while also acknowledging that open meetings may constrain committee members in expressing their views freely. If deliberation among participants is compromised by the level of openness, then that can also compromise the quality of the recommendations produced. It would therefore be interesting if Oortwijn et al could further expand on how the variation in practice observed in their study correspond to the EDP’s notion of the ideal level of transparency or if such an ‘ideal’ level can be defined from their point of view.

 Another trade-off exists between securing optimal level of inclusiveness and managing stakeholders’ interests in the process. Oortwijn et al emphasize that stakeholders with direct claim on the health technology should not be members of the advisory committee but rather be engaged through stakeholder participation or other means. While this is a sound justification for excluding industry interests, the approach risks exclusion of key voices, for example if the health technology in question is especially beneficial to marginalized populations who typically do not engage in policy processes like these. The quality of deliberation may be insufficient if their views, experiences and interests are poorly represented in the process by other civil society representatives. In future guidance about the features and implementation of EDPs, greater attention to assessment of these trade-offs and empirical insight to how these are dealt with by HTA agencies can prove valuable.

## Procedural Scope

 UHC is a redistributive concept.^9^ Decisions shaping progress towards UHC is debated and contested in a wide range of political arenas and deliberative spaces among people whose interests and values conflict. The extent to which these spaces are conducive to inclusive, open and respectful deliberation about healthcare priorities matters for fairness and legitimacy. In this context, EDPs can be seen as an attempt to “open up” the technocratic nature of structured HTA-processes to enable inclusion of value-laden disagreements in the decision-making process about health benefit package design, for example by strengthening transparency and increasing the scope for public involvement. Yet if the goal is to promote legitimacy and associated values like trust in health benefit design decisions, then the scope of processes examined must be wider than the steps involved in producing and using HTAs. Therefore, it might be prudent not to tie the legitimacy of health benefit package decisions strictly to HTA-driven processes but also the quality of deliberation in political arenas and processes where questions about health benefits are debated. In future country-level empirical assessments, it can be valuable to examine how the stepwise approach envisaged for EDPs align with the scope for public participation and inclusiveness set forth by the country’s legislative, institutional and political framework—aspects that several of the authors themselves have called into attention.^14^ For example, in Thailand, the use of annual public hearings with healthcare providers, patients and local healthcare administrators is mandated by the country’s National Health Security Act of 2002. It is integral to the perceived legitimacy of decisions shaping Thailand’s Universal Coverage Scheme, and integrated with the EDP-like procedures used by the country to determine health benefits.^15^

## Conclusion

 The EDP has evolved and contributed to significant progress in inspiring fair and legitimate health benefit design decisions in a wide range of settings. The systematic and stepwise approach holds potential for promoting decision-making processes that meet key features of procedural fairness, thereby promoting legitimacy of difficult, value-laden decisions where disagreements are likely to persist. These decision-making features have broad appeal, including to decision-making processes shaping other areas of health financing, including revenue generation and pooling. At the same time, prudence is needed when evaluating fairness and legitimacy on the basis of structured and step-wised approaches envisaged by EDPs, and that investments in EDPs is efficiently deployed to enhance the pre-existing legislative, institutional and political framework that exist to promote fair and legitimate healthcare decisions.

## Ethical issues

 Not applicable.

## Competing interests

 Author declares that he has no competing interests.

## Author’s contribution

 UG is the single author of the paper.

## Funding

 This work was supported by the Research Council of Norway through its funding for the project Supporting inclusive and accountable health systems decisions in Ghana and Kenya for universal health coverage (SUPPORT-SYSTEMS, project no. 316145).
